# Hexa­aqua­bis­[3,5-bis­(hy­droxy­imino)-1-methyl-2,4,6-trioxo­cyclo­hexa­nido-κ^2^ *N* ^3^,*O* ^4^]barium tetrahydrate

**DOI:** 10.1107/S1600536813027761

**Published:** 2013-10-16

**Authors:** Nguyen Dinh Do, Olga Kovalchukova, Adam Stash, Svetlana Strashnova

**Affiliations:** a Hanoi University of Mining and Geology, Dong Ngac, Tu Liem, Ha Noi, Vietnam; b Peoples’ Friendship University of Russia, 6, Miklukho-Mallaya, 117198 Moscow, Russian Federation; c Karpov Institute of Physical Chemistry, 10, Vorontsovo Pole, 105064 Moscow, Russian Federation

## Abstract

In the title compound, [Ba(C_7_H_5_N_2_O_5_)_2_(H_2_O)_6_]·4H_2_O, the Ba^2+^ cation lies on a twofold rotation axis and is ten-coordinated by two 3,5-bis­(hy­droxy­imino)-1-methyl-2,4,6-trioxo­cyclo­hexa­n­ide oxo O atoms [Ba—O = 2.8715 (17) Å], two hy­droxy­imino N atoms [Ba—N = 3.036 (2) Å], and six water mol­ecules [Ba—O = 2.847 (2), 2.848 (2), and 2.880 (2) Å]. The 3,5-bis­(hy­droxy­imino)-1-methyl-2,4,6-trioxo­cyclo­hexa­nide monoanions act in a bidentate chelating manner, coordinating through an N atom of the non-deprotonated hy­droxy­imino group and an O atom of the neighboring oxo group. Two lattice water mol­ecules are located in the cavities of the framework and are involved in hydrogen bonding to O atoms of one of the coordinating water mol­ecules and the O atom of a keto group of the ligand. As a result, a three-dimensional network is formed.

## Related literature

For the synthesis and crystal structure of sodium 3,5-bis­(hy­droxy­imino)-1-methyl-2,4,6- trioxo­cyclo­hexa­nide, see: Kovalchukova *et al.* (2012 ▶). For related structures of metal complexes with 1,2-benzo(naphto)quinone-1-oximes, see: Chakravorty (1974 ▶); Charalambous *et al.* (1993 ▶, 1995 ▶, 1996 ▶); Adatia *et al.* (1996 ▶); Basu & Chakravorty (1992 ▶); McPartlin (1973 ▶); Djinovic *et al.* (1992 ▶); Liu *et al.* (2010 ▶). For applications of related complexes as catalysts, see: Gharah *et al.* (2009 ▶).
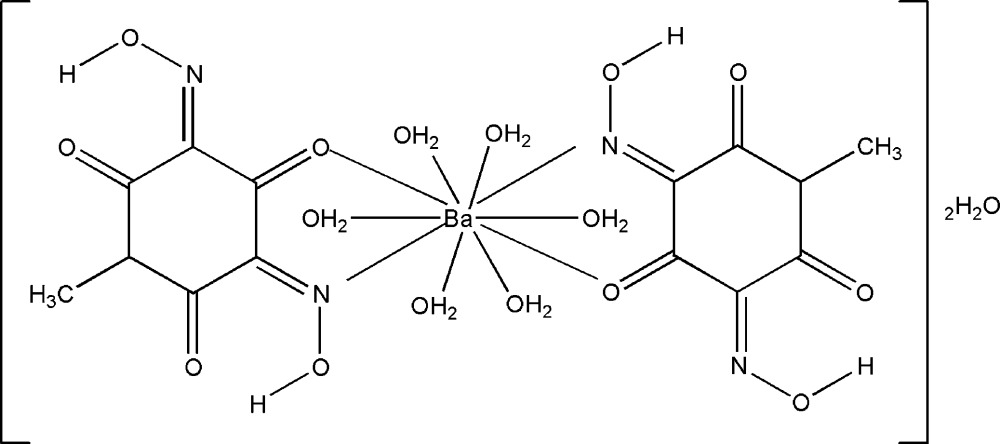


## Experimental

### 

#### Crystal data

[Ba(C_7_H_5_N_2_O_5_)_2_(H_2_O)_6_]·4H_2_O
*M* *_r_* = 711.76
Monoclinic, 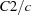

*a* = 17.235 (3) Å
*b* = 6.736 (1) Å
*c* = 23.074 (5) Å
β = 108.61 (3)°
*V* = 2538.7 (9) Å^3^
*Z* = 4
Mo *K*α radiation
μ = 1.66 mm^−1^
*T* = 293 K
0.33 × 0.12 × 0.07 mm

#### Data collection

Enraf–Nonius CAD-4 diffractometer
Absorption correction: part of the refinement model (Δ*F*) (Walker & Stuart, 1983 ▶) *T*_min_ = 0.370, *T*_max_ = 0.780
2429 measured reflections
2346 independent reflections
1755 reflections with *I* > 2σ(*I*)
*R*_int_ = 0.019
3 standard reflections every 60 min intensity decay: none

#### Refinement

*R*[*F* ^2^ > 2σ(*F* ^2^)] = 0.018
*wR*(*F* ^2^) = 0.048
*S* = 0.98
2346 reflections
216 parameters
17 restraints
H atoms treated by a mixture of independent and constrained refinement
Δρ_max_ = 0.47 e Å^−3^
Δρ_min_ = −0.90 e Å^−3^

### 

Data collection: *CAD-4-PC* (Enraf–Nonius, 1993 ▶); cell refinement: *CAD-4-PC*; data reduction: *CAD-4-PC*; program(s) used to solve structure: *SHELXS97* (Sheldrick, 2008 ▶); program(s) used to refine structure: *SHELXL97* (Sheldrick, 2008 ▶); molecular graphics: *SHELXTL* (Sheldrick, 2008 ▶); software used to prepare material for publication: *CIFTAB97* (Sheldrick, 2008 ▶) and *SHELXL97*.

## Supplementary Material

Crystal structure: contains datablock(s) I, global. DOI: 10.1107/S1600536813027761/bv2225sup1.cif

Structure factors: contains datablock(s) I. DOI: 10.1107/S1600536813027761/bv2225Isup2.hkl

Additional supplementary materials:  crystallographic information; 3D view; checkCIF report

## Figures and Tables

**Table 1:** Hydrogen-bond geometry (Å, °)

*D*—H⋯*A*	*D*—H	H⋯*A*	*D*⋯*A*	*D*—H⋯*A*
O21—H21⋯O3	0.86 (3)	1.70 (3)	2.476 (3)	150 (4)
O61—H61⋯O5	0.86 (3)	1.64 (3)	2.469 (2)	160 (4)
O11—H111⋯O3^i^	0.85 (1)	1.99 (1)	2.827 (2)	167 (4)
O11—H112⋯O12^ii^	0.84 (1)	2.15 (3)	2.854 (3)	142 (4)
O12—H121⋯O14^iii^	0.84 (1)	1.90 (1)	2.739 (3)	172 (4)
O12—H122⋯O11^iv^	0.84 (1)	2.17 (3)	2.839 (3)	137 (3)
O13—H131⋯O14	0.85 (1)	2.13 (1)	2.957 (3)	165 (4)
O13—H132⋯O15^v^	0.85 (1)	1.93 (1)	2.766 (3)	169 (4)
O14—H141⋯O13^vi^	0.85 (1)	1.99 (1)	2.835 (3)	173 (4)
O15—H151⋯O5^vii^	0.85 (1)	1.94 (1)	2.789 (3)	177 (4)

Symmetry codes: (i) 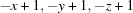; (ii) 
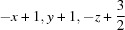
; (iii) 
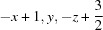
; (iv) 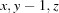; (v) 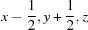; (vi) 
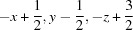
; (vii) 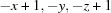.

**Fig. 1.**
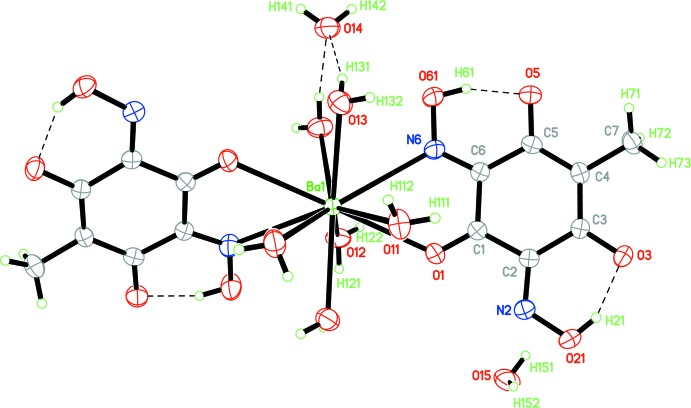
ORTEP view of C14H30BaN4O20 with atom labeling scheme (displacement ellipsoids are drawn at the 50% probability level for non-hydrogen atoms). Hydrogen bonds shown as dashed lines.

**Fig. 2.**
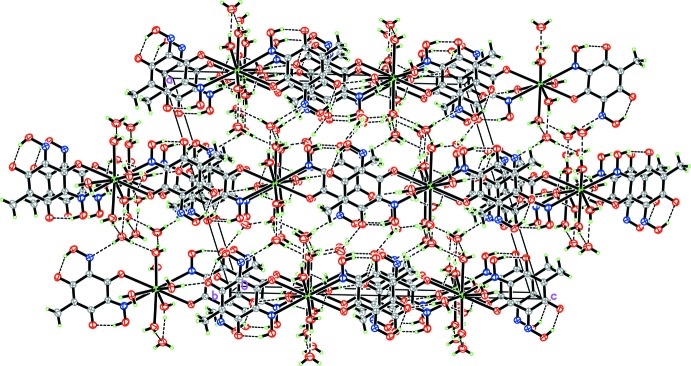
Molecular packing in the crystal of the complex along the crystallographic axis b. Hydrogen bonds shown as dashed lines.

## supplementary crystallographic information

### 1. Comment

1,2-Quinone monooximes (2-nitrosophenols) are good chelating agents which form stable colored complexes with a wide range of metal ions (Chakravorty, 1974; Charalambous *et al.*, 1996; Djinovic *et al.*, 1992; Liu *et al.*, 2010). Increasing attention is also being devoted to these complexes in recent years because of their applications in organic synthesis (Gharah *et al.*, 2009). Sodium 3,5-bis(hydroxyimino) -1-methyl-2,4,6-trioxocyclohexanide has been recently isolated as the only product of the reaction of nitrosation of methylphloroglucinol, and its crystal and molecular structure was reported (Kovalchukova *et al.*, 2012). In the present paper we report the crystal and molecular structure of barium hexaaqua bis (3,5-bis(hydroxyimino)-1-methyl-2,4,6- trioxocyclohexanide) dihydrate, C_14_H_30_BaN_4_O_20_. The Ba cation of the reported structure lies on a center of inversion, and is ten-coordinated. It is surrounded by two oxo O atoms of 3,5-bis(hydroxyimino)-1-methyl-2,4,6- trioxocyclohexanide species [Ba—O = 2.8715 (17) Å], two hydroxyimino N atoms [Ba—N = 3.036 (2) Å], and six water molecules [Ba—O = 2.847 (2), 2.848 (2), and 2.880 (2) Å]. The 3,5-bis(hydroxyimino)-1-methyl-2,4,6- trioxocyclohexanide mono anions act as bidentate chelating coordinated through an N-atom of the non-deprotonated hydroxyimino group and an O-atom of the neighboring oxo-group. The conjugation in the chelate ring is observed in the equalizing of the chemical bonds [C1—O1 = 1.219 (3), C6—N6 = 1.299 (3), N6—O61 = 1.352, C1—C6 1.482 Å]. There are two intramolecular hydrogen bonds in the 3,5-bis(hydroxyimino)-1-methyl-2,4,6- trioxocyclohexanide anion adjusting the hydroxyimino H atoms with the neighboring O oxo atoms. The coordinated water molecules are involved in O—H···O hydrogen bonds with the O atoms of the organic anion. Two lattice water molecules are located in the cavities of the framework and involved in hydrogen bonding to O atoms of one of the coordinated H_2_O and the O atom of an oxo group of the ligand. As a result, a three-dimensional network is formed. The described structure is in accordance with the other known structures of complexes of 1,2-benzo(naphto) quinone -1-oximes with the alkaline (Charalambous *et al.*, 1993,1995; Adatia *et al.*, 1996) and transition metals (Basu and Chakravorty, 1992; Charalambous *et al.*, 1996; McPartlin, 1973) where the formation of monomeric complexes with the bidentate chelating coordination mode of the ligands was observed. The only one difference is in the nature of the hydroxyimino group which is not ionized in the structure reported here. From the other side, the 3,5-bis(hydroxyimino)- 1-methyl-2,4,6-trioxocyclohexanide anion in the sodium salt (Kovalchukova *et al.*, 2012) acts as m-1,2,2,3 polydentate bridging ligands were both N and O hydroxyimino atoms take part in coordination, and one of the O oxo coordinated atoms forms a bifurcated bond.

### 2. Experimental

Single crystals of C_14_H_30_BaN_4_O_20_ were grown by the slow evaporation of the ethanol solution of the 1-to-1 molar mixture of barium nitrate and sodium 3,5-bis(hydroxyimino)-1-methyl-2,4,6-trioxo cyclohexanide.

### 3. Refinement

The structure of of C_14_H_30_BaN_4_O_20_ was solved by direct methods and all non-hydrogen atoms were located and refined in anisotropically. All the hydrogen atoms were located in difference electron density syntheses and included in refinement with fixed parameters. The O-H distances in the hydroxy groups were constrained at 0.84 Å. The O-H distances and H-O-H angles in the water molecules were constrained. A riding model was used in the refinement of H atoms of the methyl group.

### Crystal data

**Table:**

[Ba(C_7_H_5_N_2_O_5_)_2_(H_2_O)_6_]·4H_2_O	*F*(000) = 1432
*M**_r_* = 711.76	*D*_x_ = 1.862 Mg m^−^^3^
Monoclinic, *C*2/*c*	Mo *K*α radiation, λ = 0.71073 Å
Hall symbol: -C 2yc	Cell parameters from 25 reflections
*a* = 17.235 (3) Å	θ = 9.2–11.7°
*b* = 6.736 (1) Å	µ = 1.66 mm^−^^1^
*c* = 23.074 (5) Å	*T* = 293 K
β = 108.61 (3)°	Plate, red
*V* = 2538.7 (9) Å^3^	0.33 × 0.12 × 0.07 mm
*Z* = 4	

### Data collection

**Table:**

Enraf–Nonius CAD-4 diffractometer	1755 reflections with *I* > 2σ(*I*)
Radiation source: fine-focus sealed tube	*R*_int_ = 0.019
β-filter monochromator	θ_max_ = 25.5°, θ_min_ = 1.9°
ω/2θ scans	*h* = 0→20
Absorption correction: part of the refinement model (Δ*F*) (Walker & Stuart, 1983)	*k* = −8→0
*T*_min_ = 0.370, *T*_max_ = 0.780	*l* = −27→26
2429 measured reflections	3 standard reflections every 60 min
2346 independent reflections	intensity decay: none

### Refinement

**Table:**

Refinement on *F*^2^	Primary atom site location: structure-invariant direct methods
Least-squares matrix: full	Secondary atom site location: difference Fourier map
*R*[*F*^2^ > 2σ(*F*^2^)] = 0.018	Hydrogen site location: difference Fourier map
*wR*(*F*^2^) = 0.048	H atoms treated by a mixture of independent and constrained refinement
*S* = 0.98	*w* = 1/[σ^2^(*F*_o_^2^) + (0.0317*P*)^2^] where *P* = (*F*_o_^2^ + 2*F*_c_^2^)/3
2346 reflections	(Δ/σ)_max_ = 0.001
216 parameters	Δρ_max_ = 0.47 e Å^−^^3^
17 restraints	Δρ_min_ = −0.90 e Å^−^^3^

### Special details

**Table:**

Geometry. All e.s.d.'s (except the e.s.d. in the dihedral angle between two l.s. planes) are estimated using the full covariance matrix. The cell e.s.d.'s are taken into account individually in the estimation of e.s.d.'s in distances, angles and torsion angles; correlations between e.s.d.'s in cell parameters are only used when they are defined by crystal symmetry. An approximate (isotropic) treatment of cell e.s.d.'s is used for estimating e.s.d.'s involving l.s. planes.
Refinement. Refinement of *F*^2^ against ALL reflections. The weighted *R*-factor *wR* and goodness of fit *S* are based on *F*^2^, conventional *R*-factors *R* are based on *F*, with *F* set to zero for negative *F*^2^. The threshold expression of *F*^2^ > σ(*F*^2^) is used only for calculating *R*-factors(gt) *etc*. and is not relevant to the choice of reflections for refinement. *R*-factors based on *F*^2^ are statistically about twice as large as those based on *F*, and *R*- factors based on ALL data will be even larger.

### Fractional atomic coordinates and isotropic or equivalent isotropic displacement parameters (Å^2^)

**Table:**

	*x*	*y*	*z*	*U*_iso_*/*U*_eq_	
Ba1	0.5000	0.24104 (3)	0.7500	0.02124 (7)	
O1	0.56648 (10)	0.2550 (3)	0.65071 (7)	0.0332 (4)	
O3	0.54847 (10)	0.3322 (3)	0.44397 (7)	0.0317 (4)	
O5	0.32241 (10)	0.0962 (3)	0.49495 (7)	0.0349 (4)	
O11	0.48141 (12)	0.6073 (3)	0.68255 (8)	0.0377 (4)	
O12	0.59225 (10)	−0.1143 (3)	0.75770 (9)	0.0368 (4)	
O13	0.32986 (11)	0.3326 (3)	0.70959 (8)	0.0371 (4)	
O14	0.24224 (11)	−0.0492 (3)	0.70127 (9)	0.0447 (5)	
O15	0.80288 (12)	0.0550 (3)	0.60419 (10)	0.0486 (5)	
O21	0.67935 (10)	0.3795 (3)	0.52748 (8)	0.0335 (4)	
O61	0.33722 (12)	0.0432 (3)	0.60383 (8)	0.0443 (5)	
N2	0.64131 (11)	0.3306 (3)	0.56837 (9)	0.0259 (4)	
N6	0.41475 (12)	0.1133 (3)	0.61886 (9)	0.0317 (4)	
C1	0.52814 (12)	0.2383 (3)	0.59644 (9)	0.0226 (4)	
C2	0.56427 (13)	0.2839 (3)	0.54772 (10)	0.0212 (4)	
C3	0.51345 (14)	0.2780 (3)	0.48228 (10)	0.0214 (4)	
C4	0.43167 (14)	0.2147 (3)	0.46508 (9)	0.0230 (4)	
C5	0.39533 (13)	0.1576 (3)	0.50912 (10)	0.0235 (4)	
C6	0.44222 (13)	0.1663 (3)	0.57513 (10)	0.0222 (4)	
C7	0.38032 (15)	0.2124 (4)	0.39871 (10)	0.0314 (5)	
H71	0.3245	0.2422	0.3951	0.038*	
H72	0.3831	0.0834	0.3818	0.038*	
H73	0.4005	0.3101	0.3769	0.038*	
H21	0.646 (2)	0.360 (7)	0.4916 (14)	0.077 (4)*	
H61	0.321 (2)	0.050 (6)	0.5645 (14)	0.077 (4)*	
H111	0.470 (2)	0.606 (6)	0.6438 (5)	0.077 (4)*	
H112	0.4404 (16)	0.664 (6)	0.6874 (16)	0.077 (4)*	
H121	0.6431 (8)	−0.096 (6)	0.7668 (16)	0.077 (4)*	
H122	0.582 (2)	−0.198 (5)	0.7293 (14)	0.077 (4)*	
H131	0.302 (2)	0.228 (4)	0.7002 (17)	0.077 (4)*	
H132	0.315 (2)	0.399 (5)	0.6766 (10)	0.077 (4)*	
H141	0.217 (2)	−0.083 (6)	0.7256 (12)	0.077 (4)*	
H142	0.2137 (19)	−0.102 (6)	0.6680 (9)	0.077 (4)*	
H151	0.7660 (19)	0.008 (5)	0.5737 (12)	0.077 (4)*	
H152	0.794 (3)	0.1784 (18)	0.6008 (18)	0.077 (4)*	

### Atomic displacement parameters (Å^2^)

**Table:**

	*U*^11^	*U*^22^	*U*^33^	*U*^12^	*U*^13^	*U*^23^
Ba1	0.02168 (10)	0.02131 (10)	0.02071 (9)	0.000	0.00676 (6)	0.000
O1	0.0286 (8)	0.0467 (10)	0.0227 (7)	−0.0042 (8)	0.0059 (6)	−0.0036 (8)
O3	0.0325 (9)	0.0387 (9)	0.0265 (8)	−0.0059 (8)	0.0131 (7)	0.0014 (7)
O5	0.0256 (8)	0.0448 (10)	0.0327 (9)	−0.0101 (8)	0.0070 (7)	−0.0014 (8)
O11	0.0491 (11)	0.0352 (10)	0.0281 (8)	0.0063 (8)	0.0116 (8)	0.0013 (7)
O12	0.0292 (8)	0.0334 (9)	0.0473 (10)	−0.0028 (8)	0.0114 (8)	−0.0080 (8)
O13	0.0344 (9)	0.0388 (10)	0.0347 (9)	0.0039 (8)	0.0062 (8)	0.0012 (8)
O14	0.0308 (9)	0.0582 (13)	0.0432 (10)	−0.0059 (9)	0.0092 (8)	0.0087 (10)
O15	0.0353 (10)	0.0522 (12)	0.0471 (11)	−0.0040 (10)	−0.0025 (8)	0.0100 (10)
O21	0.0273 (8)	0.0412 (10)	0.0351 (9)	−0.0068 (8)	0.0145 (7)	0.0002 (8)
O61	0.0343 (9)	0.0687 (14)	0.0310 (9)	−0.0242 (10)	0.0120 (8)	−0.0031 (9)
N2	0.0267 (10)	0.0223 (9)	0.0292 (10)	−0.0008 (8)	0.0097 (8)	−0.0012 (8)
N6	0.0294 (10)	0.0372 (12)	0.0293 (10)	−0.0115 (9)	0.0105 (8)	−0.0031 (9)
C1	0.0246 (10)	0.0187 (9)	0.0241 (9)	0.0008 (9)	0.0073 (8)	−0.0011 (9)
C2	0.0235 (10)	0.0137 (11)	0.0263 (10)	0.0013 (8)	0.0078 (8)	−0.0025 (8)
C3	0.0269 (10)	0.0138 (11)	0.0242 (9)	0.0021 (8)	0.0092 (8)	0.0000 (8)
C4	0.0293 (11)	0.0156 (11)	0.0226 (10)	0.0022 (8)	0.0064 (8)	0.0005 (8)
C5	0.0229 (11)	0.0180 (10)	0.0277 (11)	−0.0004 (9)	0.0054 (8)	−0.0018 (9)
C6	0.0250 (11)	0.0174 (9)	0.0244 (10)	0.0001 (9)	0.0082 (9)	−0.0011 (8)
C7	0.0352 (12)	0.0301 (13)	0.0245 (10)	−0.0031 (9)	0.0033 (9)	0.0003 (9)

### Geometric parameters (Å, º)

**Table:**

Ba1—O13	2.8467 (19)	O15—H151	0.846 (10)
Ba1—O13^i^	2.8467 (19)	O15—H152	0.845 (10)
Ba1—O12	2.8475 (19)	O21—N2	1.350 (3)
Ba1—O12^i^	2.8475 (19)	O21—H21	0.86 (3)
Ba1—O1^i^	2.8715 (17)	O61—N6	1.354 (3)
Ba1—O1	2.8715 (17)	O61—H61	0.86 (3)
Ba1—O11^i^	2.8799 (19)	N2—C2	1.298 (3)
Ba1—O11	2.8799 (19)	N6—C6	1.294 (3)
Ba1—N6	3.036 (2)	C1—C2	1.481 (3)
Ba1—N6^i^	3.036 (2)	C1—C6	1.485 (3)
O1—C1	1.220 (3)	C2—C3	1.485 (3)
O3—C3	1.273 (3)	C3—O3	1.273 (3)
O5—C5	1.263 (3)	C3—C4	1.403 (3)
O11—H111	0.851 (10)	C4—C5	1.407 (3)
O11—H112	0.841 (10)	C4—C7	1.504 (3)
O12—H121	0.842 (10)	C5—O5	1.263 (3)
O12—H122	0.841 (10)	C5—C6	1.480 (3)
O13—H131	0.847 (10)	C7—H71	0.9600
O13—H132	0.847 (10)	C7—H72	0.9600
O14—H141	0.851 (10)	C7—H73	0.9600
O14—H142	0.849 (10)		
			
O13—Ba1—O13^i^	154.98 (9)	Ba1—O11—H111	121 (3)
O13—Ba1—O12	134.33 (6)	Ba1—O11—H112	106 (3)
O13^i^—Ba1—O12	70.45 (6)	H111—O11—H112	103 (2)
O13—Ba1—O12^i^	70.45 (6)	Ba1—O12—H121	114 (3)
O13^i^—Ba1—O12^i^	134.33 (5)	Ba1—O12—H122	123 (3)
O12—Ba1—O12^i^	65.59 (7)	H121—O12—H122	104 (2)
O13—Ba1—O1^i^	67.83 (5)	Ba1—O13—H131	111 (3)
O13^i^—Ba1—O1^i^	111.29 (5)	Ba1—O13—H132	113 (3)
O12—Ba1—O1^i^	109.55 (5)	H131—O13—H132	103 (2)
O12^i^—Ba1—O1^i^	73.76 (5)	H141—O14—H142	102 (2)
O13—Ba1—O1	111.30 (5)	H151—O15—H152	103 (2)
O13^i^—Ba1—O1	67.83 (5)	N2—O21—H21	108 (3)
O12—Ba1—O1	73.76 (5)	N6—O61—H61	102 (3)
O12^i^—Ba1—O1	109.55 (5)	C2—N2—O21	118.06 (19)
O1^i^—Ba1—O1	176.25 (8)	C6—N6—O61	118.25 (19)
O13—Ba1—O11^i^	85.23 (6)	C6—N6—Ba1	121.19 (14)
O13^i^—Ba1—O11^i^	73.27 (6)	O61—N6—Ba1	118.58 (13)
O12—Ba1—O11^i^	136.29 (6)	O1—C1—C2	122.60 (19)
O12^i^—Ba1—O11^i^	135.85 (5)	O1—C1—C6	121.73 (19)
O1^i^—Ba1—O11^i^	62.92 (5)	C2—C1—C6	115.65 (18)
O1—Ba1—O11^i^	113.53 (5)	N2—C2—C1	113.59 (19)
O13—Ba1—O11	73.27 (6)	N2—C2—C3	125.6 (2)
O13^i^—Ba1—O11	85.23 (6)	C1—C2—C3	120.79 (19)
O12—Ba1—O11	135.85 (5)	O3—C3—C4	123.2 (2)
O12^i^—Ba1—O11	136.29 (5)	O3—C3—C4	123.2 (2)
O1^i^—Ba1—O11	113.53 (5)	O3—C3—C2	116.23 (19)
O1—Ba1—O11	62.92 (5)	O3—C3—C2	116.23 (19)
O11^i^—Ba1—O11	62.11 (7)	C4—C3—C2	120.62 (19)
O13—Ba1—N6	67.33 (6)	C3—C4—C5	121.15 (19)
O13^i^—Ba1—N6	120.54 (6)	C3—C4—C7	120.2 (2)
O12—Ba1—N6	84.66 (6)	C5—C4—C7	118.7 (2)
O12^i^—Ba1—N6	67.46 (6)	O5—C5—C4	122.6 (2)
O1^i^—Ba1—N6	127.95 (5)	O5—C5—C4	122.6 (2)
O1—Ba1—N6	53.38 (5)	O5—C5—C6	116.8 (2)
O11^i^—Ba1—N6	135.75 (6)	O5—C5—C6	116.8 (2)
O11—Ba1—N6	76.65 (6)	C4—C5—C6	120.64 (19)
O13—Ba1—N6^i^	120.54 (6)	N6—C6—C5	125.2 (2)
O13^i^—Ba1—N6^i^	67.33 (6)	N6—C6—C1	113.97 (19)
O12—Ba1—N6^i^	67.46 (6)	C5—C6—C1	120.87 (19)
O12^i^—Ba1—N6^i^	84.66 (6)	C4—C7—H71	109.5
O1^i^—Ba1—N6^i^	53.38 (5)	C4—C7—H72	109.5
O1—Ba1—N6^i^	127.96 (5)	H71—C7—H72	109.5
O11^i^—Ba1—N6^i^	76.65 (6)	C4—C7—H73	109.5
O11—Ba1—N6^i^	135.76 (6)	H71—C7—H73	109.5
N6—Ba1—N6^i^	147.08 (8)	H72—C7—H73	109.5
C1—O1—Ba1	126.33 (14)		

Symmetry code: (i) −*x*+1, *y*, −*z*+3/2.

### Hydrogen-bond geometry (Å, º)

**Table:**

*D*—H···*A*	*D*—H	H···*A*	*D*···*A*	*D*—H···*A*
O21—H21···O3	0.86 (3)	1.70 (3)	2.476 (3)	150 (4)
O61—H61···O5	0.86 (3)	1.64 (3)	2.469 (2)	160 (4)
O11—H111···O3^ii^	0.85 (1)	1.99 (1)	2.827 (2)	167 (4)
O11—H112···O12^iii^	0.84 (1)	2.15 (3)	2.854 (3)	142 (4)
O12—H121···O14^i^	0.84 (1)	1.90 (1)	2.739 (3)	172 (4)
O12—H122···O11^iv^	0.84 (1)	2.17 (3)	2.839 (3)	137 (3)
O13—H131···O14	0.85 (1)	2.13 (1)	2.957 (3)	165 (4)
O13—H132···O15^v^	0.85 (1)	1.93 (1)	2.766 (3)	169 (4)
O14—H141···O13^vi^	0.85 (1)	1.99 (1)	2.835 (3)	173 (4)
O15—H151···O5^vii^	0.85 (1)	1.94 (1)	2.789 (3)	177 (4)

Symmetry codes: (i) −*x*+1, *y*, −*z*+3/2; (ii) −*x*+1, −*y*+1, −*z*+1; (iii) −*x*+1, *y*+1, −*z*+3/2; (iv) *x*, *y*−1, *z*; (v) *x*−1/2, *y*+1/2, *z*; (vi) −*x*+1/2, *y*−1/2, −*z*+3/2; (vii) −*x*+1, −*y*, −*z*+1.

## References

[bb1] Adatia, T., Chakrabarti, J., Charalambous, J., Carugo, O. & Castallani, C. B. (1996). *Polyhedron*, **15**, 1331–1338.

[bb2] Basu, P. & Chakravorty, A. (1992). *J. Chem. Soc. Chem. Commun.* pp. 809–810.

[bb3] Chakravorty, A. (1974). *Coord. Chem. Rev.* **13**, 1–46.

[bb4] Charalambous, J., Fogg, P. G. T., Gaganatsou, P. & Hendrick, K. (1993). *Polyhedron*, **12**, 879–882.

[bb5] Charalambous, J., Raghvani, D. V., Carugo, O., Castallani, C. B. & Sardone, N. (1996). *Polyhedron*, **15**, 803–808.

[bb6] Charalambous, J., Rees, R. G. & Thomas, T. A. (1995). *Polyhedron*, **14**, 2541–2556.

[bb7] Djinovic, K., Carugo, O. & Castellani, C. B. (1992). *Inorg. Chim. Acta*, **202**, 59–65.

[bb8] Enraf–Nonius (1993). *CAD-4-PC* Enraf–Nonius, Delft, The Netherlands.

[bb9] Gharah, N., Chakraborty, S., Mukherjee, A. K. & Battacharya, R. (2009). *Inorg. Chim. Acta*, **362**, 1089–1100.

[bb10] Kovalchukova, O. V., Dinh Do, N., Stash, A., Bel’sky, V., Strashnov, P., Alafinov, A., Volyansky, O., Strashnova, S. B. & Kobrakov, K. E. (2012). *Cryst. Struct. Theory Appl.* **1**, 46–51.

[bb11] Liu, Y.-N., Liang, W.-Z., Sang, X.-G., Huo, Y.-Q., Lap, S.-T., Yung, K.-F. & Liu, X.-X. (2010). *Inorg. Chim. Acta*, **363**, 949–956.

[bb12] McPartlin, M. (1973). *Inorg. Nucl. Chem. Lett.* **9**, 1207–1210.

[bb13] Sheldrick, G. M. (2008). *Acta Cryst.* A**64**, 112–122. 10.1107/S0108767307043930 18156677

[bb14] Walker, N. & Stuart, D. (1983). *Acta Cryst.* A**39**, 158–166.

